# Increases in the completeness of disease records in dairy databases following changes in the criteria determining whether a record counts as correct

**DOI:** 10.1186/1751-0147-54-71

**Published:** 2012-12-03

**Authors:** Ann-Kristina Lind, Hans Houe, Mari N Espetvedt, Cecilia Wolff, Simo Rintakoski, Peter T Thomsen

**Affiliations:** 1 Department of Large Animal Sciences, Faculty of Health and Medical Sciences, University of Copenhagen, Grønnegårdsvej 8, Copenhagen, Denmark; 2 Department of Production Animal Clinical Science, Norwegian School of Veterinary Science, P.O. Box 8146 Dep, Oslo, NO-0033, Norway; 3 Department of Clinical Sciences, Swedish University of Agricultural Sciences, P.O. Box 7054, Uppsala, SE-750 07, Sweden; 4 Department of Veterinary Biosciences, University of Helsinki, P.O. Box 66, Helsinki, FI-00014, Finland; 5 Department of Animal Science, Aarhus University, Blichers Allé 20, Tjele, DK-8830, Denmark

**Keywords:** Nordic dairy disease databases, Validation, Completeness, Farmer disease recording, Locomotor disorders

## Abstract

**Background:**

The four Nordic countries: Denmark (DK), Finland (FIN), Norway (NO) and Sweden (SE), all have national databases in which mainly records of treated animals are maintained. Recently, the completeness of locomotor disorder records in these databases has been evaluated using farmers’ recordings as a reference level. The objective of the present study was to see how previous estimates of completeness figures are affected by the criteria determining whether a recording in the database is to be judged correct. These demands included date of diagnosis and disease classification. In contrast with the previous study, a period of time between the date of disease recording in the database and by the farmer was allowed. Further, the calculations were brought to bear on individual locomotor diagnoses instead of a common locomotor disease complex code.

**Methods:**

Randomly selected dairy herds (≥ 15 cows) were invited to participate. During two 2-month periods in 2008 the farmers recorded the diseases they observed on the farm and their recordings constituted a farmer database (FD). These recordings were compared to disease recordings in the National Databases (ND). Earlier calculations of completeness for locomotor complex cases assuming an exact match on date were compared with ±7 day and ±30 day discrepancies calculated in this study.

**Results:**

The farmers in DK, FIN, NO and SE recorded 426, 147, 97 and 193 locomotor disorders, respectively. When a window of ±7 days was allowed there was a relative increase in completeness figures lying in the range of 24–100%. Further increases were minor, or non-existent, when the window was expanded to ±30 days. The same trend was seen for individual diagnoses.

**Conclusion:**

In all four of the Nordic countries a common pattern can be observed: a further increase in completeness occurs when individual locomotor diagnoses recorded by the farmer are permitted to match any locomotor diagnosis recorded in the ND. Completeness increased when both time span and different diagnoses within the locomotor complex were allowed.

## Background

On-farm primary data collection for epidemiological studies is time consuming and thus expensive. Register data offer a cost-effective alternative where they are available. Researchers’ access to data in centralised databases has given unique opportunities for epidemiologic research and there are several examples of published studies [1-3]. It is important to know how accurately register data reflect true clinical disease status. Validation studies have been undertaken recently [4-6]. However, specific guidelines setting out the criteria that must be met if a diagnosis in the database is to be classified as correct (i.e. accuracy criteria) are needed. For instance, guidelines on the allowable period of time between a disease being recorded in the dairy disease database and the true disease occurrence are needed. It also needs to be clear how closely the definition of a disease diagnosis in the database must match on-farm diagnosis, as these may vary. These elements will obviously have impact on an investigation of the validity of disease recordings in the database, but the size of the impact needs to be estimated.

Earlier findings revealed that register data on locomotor disorders in the Nordic countries, Denmark (DK), Finland (FIN), Norway (NO) and Sweden (SE), have a low completeness compared to observations made by the farmer as the referent level. Completeness figures in the range 0.17– 0.37 were found when five individual locomotor diagnoses were combined into a locomotor disorder complex [7]. The completeness was based on the requirement that a recording in the database and a recording by the farmer should have the same date recorded. However, locomotor disorders include diseases that often occur over a longer period of time [8], and therefore it can be argued that longer time spans between farmer recordings and database recordings are acceptable.

The objective of the present study was twofold: 1) to investigate whether, there is an increase in completeness when a time span of ±7 and ±30 days is permitted relative to earlier findings for exact date match between farmer recorded disease events and disease recordings. The investigation was made in four national dairy disease databases where individual diagnoses were grouped into a locomotor complex [7]; and 2) to assess completeness figures of individual (instead of grouped) locomotor diagnoses on exact date, ±7 and a ±30 days discrepancy match between farmer recorded disease events and database recordings obtained from four Nordic countries dairy disease databases.

## Materials and methods

### Selection of herds

The study population consisted of herds in the four Nordic countries with at least 15 dairy cows at the time of data sampling. The cut-off of 15 dairy cows was chosen to exclude the smallest farms in especially FIN and NO but still keep small farms to have a representative sample of these countries. In DK, NO and SE only dairy herds in milk recording were included. In FIN those herds in milk recording that participate in the disease surveillance were included. The National Databases are one part of the milk yield control or health surveillance system and they are in all countries designed to capture all medically treated animals and therefore not diseased animals that were not medically treated except for specific code addressing that there were no medical treatment given. The proportion of dairy herds participating in recording scheme was in 2007 90%, 70%, 97% and 76% in DK, FIN, NO and SE respectively [6]. The Nordic national databases have been described earlier in other publications [4-6,9-13]. The data collection was performed in parallel with three other disease complexes, clinical mastitis, metabolic- and reproductive disturbances. Results regarding these three disease complexes are presented elsewhere [6,14]. The sample size was calculated country-wise and found to be 150-200 herds in each country. Taking an expected completeness of 80% into account and average herd size, the national registered disease incidences of previous years [6,14]. A random sample of 1000, 900, 800 and 400 farmers in DK, FIN, NO and SE, respectively received an invitation to participate in the project in 2008.

### Study design

This was an observational cross-sectional study modified in that the data were sampled during two periods in 2008. The first period was 15 February–15 April, and the second period was 15 September–15 November. These sampling periods were strictly adhered to in FIN, NO and SE, but in DK the first period started and ended 2 weeks later for practical reasons.

### Data collection

Data were collected from two sources and thus fell into two databases. The first was farmer records of disease events made during the two 2-month periods in 2008. This source is referred to below as FD. The second was records extracted from the national dairy disease databases in May 2009 covering the period January 2008 until May 2009. The extraction of data from the national databases was made six months after data recording on farm to make sure that data received from the national databases were as complete as possible and any delay in reporting disease events was omitted. This source is referred to as ND. The FD recordings for locomotor disorders were entered on a sheet designed for the purpose together with clinical mastitis, metabolic disorders and reproductive disorders. For each disease category, the farmers had the option of noting clinical signs and diagnoses, and of noting any treatments undertaken by themselves, the veterinarian or the hoof trimmer. To ensure homogeneity between countries in the FD recordings, uniform instructions and information were given to the farmers. For example, the farmers were sent the same amount of reminders and used the same recording sheets. Information, recording sheets and prepaid envelopes were provided for the farmers ahead of each data collecting period. For additional details please see Lind et al. [7].

### Data management and data control

Data management, including data control and data analysis, was performed using SAS version 9.2 (SAS institute Inc., Cary, NC, USA). Herds and cows with unknown identification numbers and farmer observations recorded outside the study period were removed from FD. Hoof trimmer data obtained from ND were also deleted since FD recordings were clinical events and hoof trimmer data often include subclinical hoof disorders. Any records in FD where only a hoof trimmer attended the case were also removed from the sample (n = 4). In addition, records on heifers, calves and bulls were removed, leaving 212, 91, 51 and 124 veterinarian attended locomotor recordings in FD (Table 1) obtained from 105, 167, 179 and 129 herds in DK, FIN, NO and SE, respectively. Disease records relating to the same cow and same diagnosis within eight days were categorised as a single locomotor case, since the farmers were instructed to record each disease occurrence once only, and to note any re-treatments on the same recording sheet. The date, as stated by the farmer, on which the veterinarian attended the case was used for matching, since this is the date the veterinarian should report to the relevant central database if this date was missing, the date for when the farmer observed the diseased cow was used. FD and ND records of locomotor disorders were allocated to five individual diagnostic classes (Table 1). FD was adjusted to reflect cases not recorded by the farmer (FD) but recorded in ND, since such observations indicate that the farmer in most cases has at least observed the diseased animal but not recorded it in FD.

**Table 1:** The number of farmer-recorded locomotor diagnoses (FD) and the number of veterinarian attended (FD vet) locomotor diagnoses during two 2-month periods in 2008 in four Nordic countries, and the number of recorded locomotor diagnoses from the National Databases (ND) during the same period

**Country**	**Diagnosis**	**FD**	**FD vet**	**ND**	**FD-/ND+**
Denmark	Arthritis	20	20	8	8
	Laminitis	3	3	0	0
	Dermatitis	110	47	103	145
	Hoof abscess/sole ulcer	183	109	0	0
	Lameness other	54	33	82	95
	Disease events without diagnosis	38			
	Total	408	212	193	
Finland	Arthritis	16	16	21	16
	Laminitis	11	6	6	6
	Dermatitis	4	2	6	2
	Hoof abscess/sole ulcer	18	17	2	17
	Lameness other	62	50	23	50
	Disease events without diagnosis	29			
	Total	140	91	58	
Norway	Arthritis	5	5	4	5
	Laminitis	12	9	5	9
	Dermatitis	12	12	0	12
	Hoof abscess/sole ulcer	14	10	0	10
	Lameness other	27	15	17	15
	Disease events without diagnosis	8			
	Total	78	51	26	
Sweden	Arthritis	17	17	2	17
	Laminitis	3	3	5	3
	Dermatitis	13	8	0	8
	Hoof abscess/sole ulcer	48	37	14	37
	Lameness other	80	59	20	59
	Disease events without diagnosis	26			
	Total	187	124	41	

The FD-/ND + is disease events recorded in the ND but not recorded in the FD The disease events were recorded from 105, 167, 179 and 129 herds in Denmark, Finland, Norway and Sweden respectively and clinical signs and/or a diagnosis were given by the farmer.

### Data analysis

Completeness in ND (Formula 1) was calculated for all farmer-recorded locomotor disorders where farmers indicated that a veterinarian had been involved. To enable the completeness figures in the four countries to be compared, exact 95% confidence intervals for a proportion [15] were calculated for all completeness figures. Further, the completeness figures were calculated when adjustments had been made to reflect non-recorded farmer cases (FD) but recorded in ND, (Formula 2) (Table 2).

**Table 2:** Two-by-two tabulation illustrating the way recordings in a National Database (ND) compare with recordings by the farmer (FD)

	**FD +**	**FD -**	**Total**
ND +	a (FD+/ND+)	b (FD-/ND+)	a + b
ND -	c (FD+/ND-)	d* (FD-/ND-)	c + d
Total	a + c	b + d	

The table is used to calculate the completeness of ND recordings as compared with FD recordings.

*d could not be assessed.

Completeness was calculated as:

(1) $$\text{Completeness}=\text{Pr}\left\{T+\;|D+\right\}=a/\left(a+c\right)$$

The adjusted completeness was calculated as:

(2) $$\text{Completeness}=\text{Pr}\left\{T+|D+\right\}=\left(b,+,a\right)/\left(a,+,b,+,c\right)$$

Where T + are disease events recorded in the ND and D + in formula (1) are events observed by farmers. D + in formula (2) includes disease events observed by the farmer and also includes the non-farmer recorded disease events registered in the ND. The a is disease occurrence that is recorded in both the FD and the ND (FD+/ND+), b is disease occurrences not recorded in the FD but registered in the ND (FD-/ND+) and c is disease occurrences recorded in the FD but not in the ND (FD+/ND-).

### Matching disease recordings in FD and ND

The following are the criteria for match based on locomotor disease complex (Table 3):

A. Exact date match within complex was defined as a match in both the FD and the ND for the same country, herd and cow identification in the locomotor complex on an exact date [7]. The individual diagnoses within the locomotor complex in the FD were matched with any locomotor diagnosis within the locomotor complex in the ND.

B. Same as A but allowing a ±7 day discrepancy match.

C. Same as A but allowing a ±30 day discrepancy match.

**Table 3:** Completeness (Com) and adjusted completeness (Com adj) with exact 95% confidence intervals (CI) were calculated for farmer-recorded data where a veterinarian attended the case compared with database recordings from four Nordic countries over two 2-month periods in 2008

	**Match**^**2**^	**Denmark**	**Finland**	**Norway**	**Sweden**
Com (95% CI)	B	0.74 (0.68;0.79)	0.39 (0.30;0.49)	0.47 (0.34;0.60)	0.21 (0.15;0.28)
Com adj (95% CI)	B	0.88 (0.82;0.92)	0.56 (0.47;0.63)	0.60 (0.48;0.71)	0.33 (0.26;0.40)
Com (95% CI)	C	0.75 (0.69;0.80)	0.39 (0.30;0.49)	0.51 (0.38;0.64)	0.21 (0.15;0.29)
Com adj (95% CI)	C	0.88 (0.83;0.92)	0.56 (0.47;0.63)	0.63 (0.51;0.73)	0.33 (0.26;0.41)

The completeness figures were calculated when allowing a locomotor complex^1^ match and a date discrepancy of ±7 (B) days and ±30 days (C).

^1^ The locomotor complex contains five locomotor categories: arthritis, laminitis, dermatitis, hoof abscess/sole ulcer, and lameness other.

^2^ Exact match between ND and FD (Match A). Com were 0.37, 0.27, 0.34 and 0.17 and Com adj were 0.64, 0.43, 0.43 and 0.26 in Denmark, Finland, Norway and Sweden, respectively [7].

The following are the criteria for match based on individual locomotor diagnoses (Table 4):

D. Match for the individual diagnosis was defined as a match in the adjusted FD with the ND for the same country, herd identification, cow identification, individual diagnosis and exact date.

E. Same as D but allowing a ±7 day discrepancy match.

F. Same as D but allowing a ±30 day discrepancy match.

**Table 4:** Adjusted completeness (Com adj) with exact 95% confidence intervals (CI) for different definitions of match; exact date (D), ±7 days discrepancy (E) or ±30 days discrepancy (F) were calculated for individual diagnoses from four Nordic countries over two 2-month periods in 2008

**Diagnose**	**Match**	**Denmark**	**Finland**	**Norway**	**Sweden**
		**Com adj (CI)**	**Com adj (CI)**	**Com adj (CI)**	**Com adj (CI)**
Arthritis	D	0.39 (0.32;0.47)	0.66 (0.47;0.80)	0.57 (0.25;0.84)	0.11 (0.03;0.33)
	E	0.50 (0.42;0.58)	0.67 (0.48;0.81)	0.63 (0.31;0.86)	0.11 (0.03;0.33)
	F	0.50 (0.42;0.58)	0.67 (0.48;0.81)	0.63 (0.31;0.86)	0.11 (0.03;0.33)
Laminitis	D	0.00 (0.00;0.12)	0.40 (0.13;0.75)	0.50 (0.24;0.76)	0.92 (0.67;0.99)
	E	0.00 (0.00:0.00)	0.45 (0.16;0.78)	0.50 (0.24;0.76)	0.92 (0.67;0.99)
	F	0.00 (0.00:0.00)	0.45 (0.16;0.78)	0.50 (0.24;0.76)	0.92 (0.67;0.99)
Dermatitis	D	0.77 (0.27;0.97)	0.78 (0.39;0.95)	0.00 (0.00;0.24)	0.00 (0.00;0.32)
	E	0.78 (0.74;0.81)	0.78 (0.39;0.95)	0.00 (0.00;0.24)	0.00 (0.00;0.32)
	F	0.78 (0.74;0.81)	0.78 (0.39;0.95)	0.00 (0.00;0.24)	0.00 (0.00;0.32)
Hoof abscess/sole ulcer	D	0.00 (0.00;0.01)	0.11 (0.03;0.31)	0.00 (0.00;0.28)	0.36 (0.23;0.52)
	E	0.00 (0.00;0.01)	0.11 (0.03;0.31)	0.00 (0.00;0.28)	0.36 (0.23;0.52)
	F	0.00 (0.00;0.01)	0.11 (0.03;0.31)	0.00 (0.00;0.28)	0.36 (0.23;0.52)
Lameness other	D	0.81 (075:0.86)	0.34 (0.24;0.46)	0.61 (0.42;0.76)	0.27 (0.18;0.38)
	E	0.87 (0.74;0.93)	0.34 (0.24;0.46)	0.61 (0.42;0.76)	0.27 (0.18;0.38)
	F	0.87 (0.74;0.93)	0.34 (0.24;0.46)	0.62 (0.44;0.77)	0.27 (0.18;0.38)

For the exact match (A) only the same periods were included in the two data sources, ND and FD. When a discrepancy of ± 7 or ± 30 days was allowed, the two 2-month period in FD was compared to the same two 2-month period in ND ± 7 or and ± 30 days, respectively.

## Results

### Match based on locomotor disease complex

When the match between FD and ND for locomotor disease complex allowed ±7 days discrepancy (B) instead of requiring an exact date match (A) 91, 13, 7 and 5 extra matches were found in DK, FIN, NO and SE, respectively. The completeness in this comparison increased from 0.37 to 0.74 (100% increase) in DK, from 0.27 to 0.39 (44%) in FIN, from 0.34 to 0.47 (38%) in NO and from 0.17 to 0.21 (24%) in SE between exact date match and ±7 days discrepancy match. For the adjusted completeness the increase was 38%, 30%, 40% and 27% in DK, FIN, NO and SE, respectively.

For the locomotor complex level the discrepancy in match between 1, 2, 3, 4, 5 and 6 days discrepancy were also calculated to investigate if it was possible to come up with an optimal cut off point for when the recorded disease are registered in the databases (data not shown). However, there were not sufficient amount of matches found so it was not possible to define a better cut-off point. For metabolic disorders there were very few extra matches beyond ±4 days (Espetvedt, 2012 - personal communication). Most of the recordings were registered in the databases within ± 7 days, since there are only minor changes in completeness between ± 7 and ± 30 days, so the ± 7 days cut-off seems to be close to optimal.

In general the adjusted completeness for the locomotor disease complex was higher than the corresponding non-adjusted completeness (Table 3). Completeness figures for ±7 days discrepancy match increased from 0.74 to 0.88, from 0.39 to 0.56, from 0.47 to 0.60 and from 0.21 to 0.33 in DK, FIN, NO and SE, respectively when adjusted completeness was used instead of non-adjusted. These figures correspond to increases of 19%, 44%, 28% and 57% in DK, FIN, NO and SE, respectively. There were no differences in completeness between the ±7 day discrepancy match and the ±30 day discrepancy match in FIN and SE, and any differences observed in DK and NO were minor (Table 3).

### Match based on individual locomotor diagnoses

The adjusted completeness figures obtained when matching on individual diagnoses increased increased only minor or not at all when comparing match D with match E. However, there are several diagnoses, where there is no difference between match D and E indicating if a right diagnosis is recorded there is a good chance that also the date will match. Adjusted completeness increased in DK when a ±7 day discrepancy match was allowed for all the individual diagnoses (i.e. match D compared to E). For example, there was a rise from 0.39 to 0.50 in the ‘arthritis’ diagnosis and a rise from 0.81 to 0.87 in the ‘lameness other’ diagnosis (Table 4).

Dermatitis in DK was associated with very low levels of completeness when an exact match was required (0.04, data not shown), even though the diagnosis was recorded more than 100 times in both FD and ND. However, when the sample is adjusted through the addition of dermatitis cases recorded in ND (but not farmer-recorded in FD) completeness increases to 0.77 (Table 4). The ‘lameness other’ diagnosis had recordings in all four countries. Here DK had the highest adjusted completeness, at 81%. By comparison completeness of 34%, 61% and 27% were obtained for FIN, NO and SE, respectively, for the D match. However, when match D was compared with E, DK was the only country where there were any further matches. In all four countries, there were only minor, or no, additional increases in completeness when F matches were allowed.

## Discussion

Completness figures for locomotor disorders increased in DK, FIN, NO and SE when an increased time span was used. This for both individual diagnoses and for the locomotor complex. Overall, completeness figures were low in all four central databases, especially the completeness figures to individual diagnosis match on exact date. The completeness of individual diagnoses increased when a discrepancy of ±7 days was allowed, but only minor, or no, changes resulted when permitted discrepancy was expanded to ±30 days. In a properly functioning database system, completeness to veterinary attended cases should approach 100%, since disease events attended by a veterinarian are expected to end up as a record in the national database. Previous validation studies have suggested that we should regard overall completeness of 90% in a disease database as high, completeness of 80-89% as fair, and completeness of 70% and below as poor [16,17]. On this basis, when allowing a ±7 days discrepancy match, completeness figures of 0.74, 0.39, 0.47 and 0.21 for veterinary attended locomotor disorder complex matches in DK, FIN, NO and SE, respectively, indicate a fair degree of completeness in DK but poor levels in the other three Nordic countries. The same applies where adjusted completeness are concerned. When comparing the validation of national databases in the Nordic region, one must remember that different classifications of completeness give rather different results. For example, while DK has a fair degree of completeness figures to locomotor complex, its completeness to individual diagnoses is as poor as that in the other three countries – suggesting that the Danish national database failed to record specific diagnoses for locomotor disorders as well as the other three countries. Further, the largest increase in matches was observed in DK when exact date match was substituted by ± 7 days discrepancy. It appears that either the disease date recordings are not exactly correct or that the Danish farmers failed to report the correct dates in FD.

Locomotor disorders often persist over a lengthy period of time [8], and consequently it can be argued that assessments of completeness to them should be based on a longer time period rather than the ±7 day discrepancy that was allowed in this study. Completeness figures were therefore also calculated for a ±30 day discrepancy match. FIN did not have any further matches when the ±30 day discrepancy was allowed, and in the other three countries completeness increased only minimally. The acceptable discrepancy will depend on the purpose of the database. If for example the aim is to evaluate a herd’s general welfare status, a difference in date of recording is not critical because the purpose is merely to count the number of welfare problems. If, on the other hand, the purpose was to calculate the time from an exposure to event a correct date would be important. It is important for the farmers, hoof trimmers, veterinarians and even those managing the databases to address the problem with locomotor registrations so as to obtain more reliable data in the future. Clear guidelines on what is acceptable are needed; lag days need to be clearly defined.

The number of diagnoses observed and the number of disease cases confirmed is quite different in the four countries. Completeness differs from one Nordic country to another, but the pattern of change is the same across all four countries: that is, completeness increases when date discrepancy is allowed. The national databases, especially the Danish one, have poor ability to identify the individual diagnoses obtained, since the completeness achieved are much lower than the completeness calculated for the locomotor complex as a whole. The other three countries generally have completeness figures lower than those in DK, but DK is the country with the greatest number of extra matches when locomotor diagnoses are permitted to match with any of the locomotor diagnoses within the locomotor complex. This means that the Danish database is less good than the other three national databases at correctly recording individual locomotor diagnoses, but better at detecting locomotor disorders as a whole. The main reason why completeness differs between complex- and individual diagnose level is the fact that for the complex diagnosis level, a diagnosise in the FD for the locomotor complex level could merge any of the five locomotor diagnoses in the ND. Whereas, for the individual diagnosis level, the individual diagnose recorded in FD must match the same individual diagnose recorded in the ND. Other reasons for the discrepancy between completeness for the locomotor complex and the completeness for individual diagnoses can only be speculated upon. The specification of the individual diagnostic codes in the four countries is quite distinct [7] and does not give an obvious explanation. In any case, it is important to set up precise criteria for the specific clinical diagnoses, whether it is ok to use locomotor complex or individual diagnoses. And it is recommended for future studies to validate the different clinical diagnosis of lameness. As the individual locomotor diagnoses have quite different aetiology, a high sensitivity and specificity of the specific diagnosis is very important. This will be necessary in future studies whether one will use the data from ND for welfare evaluations, investigations of different risk factors. For risk factors it will be necessary to look into the individual diagnoses whereas for welfare evaluations, it could be argued that it might be enough to investigate if the cow has locomotor problems or not as long as the welfare implications of the different diagnoses are believed to be the same. But still, a high validity of individual diagnosis would be a great advantage.

Adjusted FD was used due to the large amount of events that were only recorded into the ND but not into the FD. This suggests that the farmers in many cases had failed to record on the recording sheet for FD. Using the adjusted FD means that all the disease events in the ND were assumed to be correct recordings, i.e. the correctness of the ND was 100%. The use of adjusted FD is supported by validation studies for Finish [5] and Norwegian (Espetvedt, 2012 personal communication) national disease registers that show that the correctness is very high (>0.90) in both countries. Because the farmers failed to report all events that were in the ND it may be possible that they also failed to report all the non-veterinarian treated evens. All four countries had an increase in completeness when adjusting the sample, suggesting a poor compliance with the instructions given and thereby causing observation bias. It has been stated that inconsistency in recording patterns might cause bias and it is difficult to distinguish between herds with a truly low incidence and herds with a low level of disease reporting [18]. To minimize the bias, lots of effort was put into informing the farmers and reminding them to record and send in their recordings.

## Conclusion

When allowing for a ±7 days discrepancy match between farmer recordings and recordings in national databases the completeness increased compared to when no date discrepancy was allowed. Allowing a time span of ±30 days the increase was minor, or non-existent. The four Nordic countries have the same pattern of a further increase in completeness when allowing an individual locomotor diagnosis recorded by the farmer to match with any locomotor diagnosis recorded in the national database. The completeness estimates increased when allowing for both time span and different diagnoses but in general the completeness are low for locomotor disorders and especially when both date and diagnose should match completely.

## Competing interests

The authors declare that they have no competing interests.

## Authors' contribution

All authors participated in the study design. AL, SR, ME and CW was responsible for data collection in Denmark, Finland, Norway and Sweden, respectively. AL performed the statistical analysis, interpreted the data and drafted the manuscript. HH and PT participated in the statistical analyses. All authors revised the manuscript, and have read and approved the final manuscript.
